# Multifactorial examination of sex-differences in head injuries and concussions among collegiate soccer players: NCAA ISS, 2004–2009

**DOI:** 10.1186/s40621-017-0127-6

**Published:** 2017-10-25

**Authors:** Avinash Chandran, Mary J. Barron, Beverly J. Westerman, Loretta DiPietro

**Affiliations:** Department of Exercise and Nutrition Sciences, Milken Institute School of Public Health, The George Washington University, 950 New Hampshire Ave, Suite 200, Washington, DC 20052 USA

**Keywords:** Injury epidemiology, Football (soccer), Head injuries, NCAA, ISS

## Abstract

**Background:**

While head injuries and concussions are major concerns among soccer players, the multifactorial nature of head injury observations in this group remains relatively undefined. We aim to extend previous analyses and examine sex-differences in the incidence of head injuries, odds of head injuries within an injured sample, and severity of head injuries, among collegiate soccer players between 2004 and 2009.

**Methods:**

Data collected within the National Collegiate Athletic Association (NCAA) Injury Surveillance System (ISS) between the years of 2004 and 2009, were analyzed in this study. Unadjusted rate ratios (RR), compared incidence rates between categories of sex, injury mechanism, setting and competition level. We also examined sex-differences in head injury incidence rates, across categories of the other covariates. Multivariable logistic regression and negative binomial regression modeling tested the relation between sex and head injury corollaries, while controlling for contact, setting, and competition level.

**Results:**

Between 2004 and 2009, head injuries accounted for approximately 11% of all soccer-related injuries reported within the NCAA-ISS. The rate of head injuries among women was higher than among men (RR = 1.23, 95% CI = [1.08, 1.41]). The rate of head injuries due to player-to-player contact was comparable between women and men (RR = 0.95, 95% CI = [0.81, 1.11]). Whereas, the rate of injury due to contact with apparatus (ball/goal) was nearly 2.5 times higher (RR = 2.46, 95% CI = [1.76, 3.44]) and the rate due to contact with a playing surface was over two times higher (RR = 2.29, 95% CI = [1.34, 3.91]) in women than in men. In our multifactorial models, we also observed that the association between sex and head injury corollaries varied by injury mechanism.

**Conclusions:**

Sex-differences in the incidence, odds (given an injury), and severity (concussion diagnosis, time-loss) of head injuries varied by injury mechanism (player-to-player contact vs. all other mechanisms) in this sample.

## Background

It is acknowledged that soccer is the most popular sport in the world, and injuries are common among soccer players of all ages (Junge and Dvorak 2004; Waldén et al. 2011). This is particularly true of head injuries, due to the mechanics of the sport. Indeed, head injuries have been previously noted to account for between 3 and 18% (depending on age, sex, setting etc.) of all reported soccer-related injuries, and in some samples, concussions resultant of these injuries have been estimated to account for up to 11% of reported injuries (Agel et al. 2007; Conder 2015; Dick et al. 2007b; Roos et al. 2016; Yard et al. 2008). In fact, among female athletes in general, concussions are reported most often among female soccer players- with concussion incidence as high as 19.38 per 10,000 competition-related athlete-exposures and 6.31 per 10,000 overall athlete-exposures, previously reported in this group (Conder 2015; Nagahiro and Mizobuchi 2014; Zuckerman et al. 2015). Yet, the multifactorial nature of head injury observations among soccer players has not yet been examined. Although studies have linked repetitive heading to measurable brain impairment among players (Matser et al. 1998; Matser et al. 1999; Stewart et al. 2017, Virgilio et al. 2016), according to recently published literature, the most frequent mechanism associated with acute head injuries among soccer players is player-to-player contact (Andersen 2004; Comstock et al. 2015; Maher et al. 2014). It remains to be seen whether the role of injury mechanism in determining the likelihood of head injuries, and head injury corollaries is comparable among males and females.

Over 80% of all soccer-related injuries reported in the U.S., are reported among those under the age of 25 (US Consumer Product Safety Commission 2012). While this may be attributable to the higher participation proportions, it nonetheless highlights the need for further evaluation of injury observations within this group (Hulteen et al. 2017). A portion of this age cohort comprises collegiate athletes competing as part of the National Collegiate Athletic Association (NCAA). While the existing literature surrounding head injuries among collegiate players also suggests a higher risk of head injuries and concussions among female players, the studies have primarily evaluated sex-differences independent of other factors (Agel et al. 2007; Boden et al. 1998; Covassin et al. 2003; Dick et al. 2007b; Maher et al. 2014; Roos et al. 2016). So, the multifactorial nature of head injury observations has not yet been examined among collegiate soccer players either (Covassin et al. 2016; Delaney et al. 2007; Dick 2009; Roos et al. 2016; Zuckerman et al. 2015).

Currently the NCAA collects surveillance data on injuries relating to participation in 25 of their 54 sanctioned sport programs (National Collegiate Athletic Association 2016). As mentioned above, although studies continue to suggest a greater risk of head injury in female, compared with male athletes, there are no comparable results on factors that may interact with sex to influence injury risk and severity, particularly in this collegiate population (Covassin et al. 2016; Zuckerman et al. 2015). Accordingly, the purpose of this study was to examine sex-differences in the incidence of head injuries, the odds of head injuries within an injured sample, and the severity (concussion diagnosis and lost days of participation) of head injuries, from a multifactorial perspective, among collegiate soccer players. Consistent with previous literature, we hypothesized that female players would have a greater incidence of head injuries, as well as greater odds of sustaining a concussion (from a head injury), and greater time-loss due to head injury, compared with male players. In addition, we considered variability in these sex-differences across levels of several factors that have been previously linked to head injury outcomes- namely, player-to-player contact, setting, and level of competition (Covassin et al. 2003; Zuckerman et al. 2015).

## Methods

The NCAA- Injury Surveillance System (ISS) was created in 1982, and details regarding data collection techniques utilized for the 2004-2009 cohort, have been previously described (Dick et al. 2007a, Kerr et al. 2014). Briefly, certified athletic trainers associated with each participating collegiate program report injuries and supporting information about the nature of the injury on a voluntary basis directly into the NCAA-ISS. A study specifically of NCAA- ISS soccer data recorded between 2005 and 2008 reported good reliability and validity of the injury data (Kucera et al. 2011). As per the data collection guidelines of the surveillance system, a reportable injury was defined as: 1) any injury event that occurred during participation in an intercollegiate game or practice; 2) that required medical attention; and 3) restricted participation or performance for ≥1 day beyond the event (Kerr et al. 2014). Injuries to the head/face, nose, mouth, eye and ear were classified as head injuries for this analysis. Contact injuries were considered separately from non-contact injuries, and game-related injuries were separated from practice-related injuries.

To correct for varying levels of playing exposure while examining injury incidence, we calculated Athlete-Exposure (AE), defined as a single participant in a single practice session or game with any probability of injury (Kerr et al. 2014). Incidence rates (IR) were then calculated as the number of events per 1000 AEs. Sex, setting and competition-level-specific AEs were used to compute IRs for specific groups. Unadjusted rate ratios (RR), along with the 95% Wald confidence intervals (CI), compared incidence rates between sex, contact (player-to-player contact vs. all other mechanisms), setting (game vs. practice) and competition level (Division I vs. Divisions II & III). We also evaluated differences in head injury incidence rates between male and female players across categories of setting, contact, and competition level.

Then, a multivariable logistic regression model was fit with sex, contact, setting, competition level, and a first order interaction term between sex and contact, to evaluate sex-differences in the odds of head injury observation (among all soccer-related injury observations) within this sample. We then examined two indicators of head injury severity. First, a logistic regression model with the same parameters noted above, was fit to examine sex-differences in the odds of concussion diagnosis (dichotomously defined within the ISS based on symptom presentation), among all soccer-related head injury observations. Maximum likelihood parameter estimates from the logistic regression models were used to calculate adjusted odds ratios (aOR) and 95% Wald CIs. We further examined sex-differences in injury severity among the injured soccer players by regressing the count of days lost from participation due to a head injury, on the above-mentioned parameters and concussion diagnosis, using negative binomial modeling. All computations were performed using WINPEPI (PEPI-for-WINDOWS) version 11.24 and SAS 9.3 (SAS Institute, Cary NC).

## Results

Typically, an NCAA soccer season lasts between 10 and 12 weeks. During the 2004–2009 time period, an average of 62 (8%) men’s and 77 (8%) women’s programs contributed to the NCAA-ISS each year (Kerr et al. 2014). An average of 54 Division I teams (approximately 11% of teams from sponsoring institutions), 18 Division II teams (approximately 5% of teams from sponsoring institutions), and 67 Division III teams (approximately 8% of teams from sponsoring institutions) contributed data annually (Kerr et al. 2014). To preserve statistical power, we pooled Division II and III teams together in further analyses. Pooled and sex-stratified exposure data are presented in Table 1. Of the 8116 reported soccer-related injuries during this period, 860 (~11%) were head injuries. Between 2004 and 2009, the overall soccer-related injury rate was 7.5 injuries per 1000 AEs and the rate of head injury was 0.80 injuries per 1000 AEs. The sex-specific rate of soccer-related head injuries was 0.87 per 1000 AEs in women and 0.71 per 1000 AEs in men (Unadjusted RR = 1.23, 95% CI = [1.08, 1.41]).

**Table 1:** Exposure time information among men and women, by categories of competition level, setting and injury mechanism

	Men	Women	Total
Practice AEs	413,160	419,721	832,881
Game AEs	114,034	136,837	250,871
Division I AEs	215,563	251,797	467,360
Division II & III AEs	311,630	304,762	616,392
All AEs	527,194	556,558	1,083,752

**Fig. 1:**
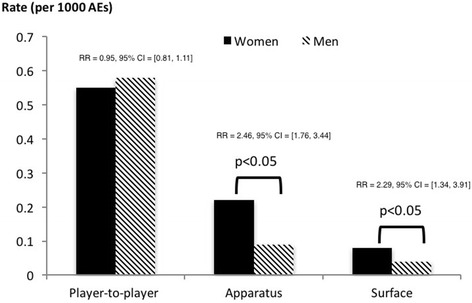
Head Injury rates by sex and by type of contact: NCAA ISS 2004/05–2008/09

The most common site of head injury was to the face (83%), followed by injuries specifically involving the nose (8%) and eye (3%). Contact of any type accounted for 98% of all head injuries, with player-to-player contact alone accounting for 71%, followed by contact with apparatus (e.g., goalposts, ball; ~20%) and contact with playing surface (~8%). The rate of player-to-player contact-related head injuries was nearly 2.5 times higher than the rate of head injuries by other mechanisms (Unadjusted RR = 2.45, 95% CI = [2.12, 2.84]). The rate of game-related head injuries was considerably higher than the rate of practice-related head injuries (Unadjusted RR = 7.29, 95% Wald CI = [6.32, 8.42]); however, level of competition (DI vs. DII/DIII) did not have a significant effect on head injury rates (Table 2). While evaluating sex-differences in head injury incidence rates, we noted comparable differences across competition levels (Division I, Divisions II & III), and no significant differences across settings (game, practice). However, sex-differences in head injury incidence rates, varied by injury mechanism (Table 2, Fig. 1). We saw significant differences in head injury incidence from non-player-to-player contact mechanisms between men and women (Table 2). In evaluating further, we noted that while the rate of head injuries due to player-to-player contact was comparable between women and men (Unadjusted RR = 0.95, 95% CI = [0.81, 1.11]), the rate of injury due to contact with apparatus was nearly 2.5 times higher (Unadjusted RR = 2.46, 95% CI = [1.76, 3.44]) and the rate due to contact with a playing surface was over two times higher (Unadjusted RR = 2.29, 95% CI = [1.34, 3.91]) in women than in men (Fig. 1).

**Table 2:** Frequencies and rates of soccer-related head injuries reported within the NCAA ISS between 2004/05 and 2008/09

Frequencies and rates (per 1000 AEs) of head injuries among men and women, by categories of competition level, setting and injury mechanism				Frequencies and rates (per 1000 AEs) of head injuries by categories of sex, competition level, setting and injury mechanism	
Explanatory variable	Men Frequency of Head Injuries (Rate per 1000 AEs)	Women Frequency of Head Injuries (Rate per 1000 AEs)	Women vs. Men Rate Ratio [95% Wald CI]	Frequency of Head Injuries (Rate per 1000 AEs)	Unadjusted Rate Ratio [95% Wald CI]
Sex					
-Women	n/a	n/a	n/a	486 (0.87)	
-Men	n/a	n/a	n/a	374 (0.71)	1.23 [1.08, 1.41]
Competition level					
-Division I	153 (0.71)	224 (0.89)	1.25 [1.02, 1.54]	377 (0.81)	
-Division II & III	221 (0.71)	262 (0.86)	1.21 [1.01, 1.45]	483 (0.78)	1.03 [0.90, 1.18]
Setting					
-Game	250 (2.19)	341 (2.49)	1.14 [0.97, 1.34]	591 (2.36)	
-Practice	124 (0.30)	145 (0.35)	1.15 [0.91, 1.46]	269 (0.32)	7.29 [6.32, 8.43]
Mechanism					
-Player-to-player contact	305 (0.58)	306 (0.55)	0.95 [0.81, 1.11]	611 (0.56)	
-All other mechanisms	69 (0.13)	180 (0.32)	2.48 [1.87, 3.26]	249 (0.23)	2.45 [2.12, 2.84]

Results from the multivariable logistic regression modeling are displayed in Tables 3 and 4. In modeling the odds of head injury within the injured sample, we observed that given an injury, sex-differences in the odds of head injury, varied by injury mechanism (player-to-player contact vs. all other mechanisms). The negative regression estimate indicated an antagonistic effect (of player-to-player contact). As such, we present stratum-specific (across levels of sex, and contact) adjusted (for setting and competition level) Odds Ratios in Table 3. Within the injured sample, while the odds of a head injury resulting from player-to-player contact emerged as significantly higher in female compared with male players (aOR = 1.23, 95% CI = [1.03, 1.47]), the odds of head injury from all other mechanisms (such as contact with apparatus or with playing surface) were nearly 2.5 times higher for women than for men (aOR = 2.41, 95% CI = [1.82, 3.20]). While evidence for this antagonistic effect was weak on the additive scale, it was strong on the multiplicative scale (Ratio of ORs = 0.508, 95% CI = [0.364, 0.711]).

**Table 3:** Varying sex-differences in odds of head injuries (given an injury), by injury mechanism (player-to-player contact)

Head injuries (*n* = 860)	Men	Women	Women vs. Men
Other mechanisms	Ref	2.41, 95% CI = [1.82, 3.20]	2.41, 95% CI = [1.82, 3.20]
Player-to-player contact mechanisms	5.99 95% CI = [4.56, 7.88]	7.35, 95% CI = [5.58, 9.69]	1.23, 95% CI = [1.03, 1.47]

Measure of variability in sex-differences on additive scale: RERI = −0.05, 95% CI = [−4.08, 3.98]

Measure of variability in sex-differences on multiplicative scale: Ratio of ORs = 0.508, 95% CI = [0.364, 0.711]

**Table 4:** Varying sex-differences in odds of concussions from head injuries, by injury mechanism (player-to-player contact)

Concussions (*n* = 582)	Men	Women	Women vs. Men
Other mechanisms	Ref	0.84, 95% CI = [0.41, 1.70]	0.84, 95% CI = [0.41, 1.70]
Player-to-player contact mechanisms	0.23, 95% CI = [0.12, 0.44]	0.49, 95% CI = [0.25, 0.95]	2.12, 95% CI = [1.50, 2.97]

Measure of variability in sex-differences on additive scale: RERI = 0.417, 95% CI = [−0.59, 1.42]

Measure of variability in sex-differences on multiplicative scale: Ratio of ORs = 2.52, 95% CI = [1.15, 5.55]

A total of 582 head injuries from this sample, resulted in concussion diagnosis (7% of all reported injuries and ~68% of head injuries). Approximately 74% of head injuries resulted in a concussion diagnosis among women, while 59% resulted in a concussion among men. In the multivariable modeling of the odds of concussion from a head injury, we again observed that sex-differences in the odds of concussion from a head injury, varied by injury mechanism. So, we present stratum-specific (across levels of sex, and contact) adjusted (for setting and competition level) Odds Ratios in Table 4. From the results presented in Table 4, we see that odds of a concussion from player-to-player contact were significantly higher in women than in men (aOR = 2.12, 95% CI = [1.50, 2.97]), after adjusting for setting and competition-level. However, the odds of concussion from other (non-player-to-player contact) mechanisms did not differ significantly between women and men (aOR = 0.84, 95% CI = [0.41, 1.70]). The evidence for this effect was weak on the additive scale, and strong on the multiplicative scale (Ratio of ORs = 2.52, 95% CI = [1.15, 5.55]).

On average among all injured players, women lost 9.21 ± 20.5 (Min: 1; Max: 367; IQR = 7) days due to a head injury, while men lost 7.95 ± 10.9 days (Min: 1; Max: 103; IQR = 7). Results from negative binomial regression modeling indicate that the expected time-loss due to head injuries within the injured cohort was lower than the expected time-loss due to all other injuries (Time-loss Ratio = 0.76, 95% CI = [0.71, 0.83]). Upon adjusting for covariates, we again observed that sex-differences in time lost from participation (due to a head injury) varied by injury mechanism. We present stratum-specific (across levels of sex, and contact) adjusted (for setting, competition level and concussion diagnosis) Time-loss Ratios in Table 5, to describe the effect. While female players lost more days from mechanisms such as contact with apparatus or with playing surface on average compared to male players (aTLR = 1.57, 95% CI = [1.20, 2.05]), sex-differences in time-loss due to player-to-player contact mechanisms were not statistically significant (aTLR = 0.93, 95% CI = [0.79, 1.08]) (Table 5). Again, the evidence for this antagonistic effect was weak on the additive scale, and strong on the multiplicative scale (Ratio of TLRs = 0.591, 95% CI = [0.433, 0.806]).

**Table 5:** Varying sex-differences in time-loss due to head injuries, by injury mechanism (player-to-player contact)

	Men	Women	Women vs. Men
Other mechanisms	Ref	1.57, 95% CI = [1.20, 2.05]	1.57, 95% CI = [1.20, 2.05]
Player-to-player contact mechanisms	1.14, 95% CI = [0.88, 1.49]	1.06, 95% CI = [0.82, 1.37]	0.925, 95% CI = [0.79, 1.08]

Measure of variability in sex-differences on additive scale: RERI = −0.65, 95% CI = [−1.56, 0.26]

Measure of variability in sex-differences on multiplicative scale: Ratio of TLRs = 0.591, 95% CI = [0.433, 0.806]

## Discussion

The most common mechanism of head injury reported among soccer players is player-to-player contact, and the results of our analyses appear consistent with the existing literature (Andersen 2004; Comstock et al. 2015; Covassin et al. 2003; Delaney et al. 2002; Maher et al. 2014; Roos et al. 2016). While repeated heading has also been examined as a causal mechanism (Gronwall and Wrightson 1975; Koutures et al. 2010; Patlak et al. 2002), a recent review of 229 published studies from 1806 to 2013 reported that player-to-player contact was the most frequent injury mechanism for both men and women (Maher et al. 2014). We observed that 71% of head injuries in our sample were due to player-to-player contact, and that the rate of head injuries due to such contact was nearly 2.5 times higher than the rate due to other mechanisms. In the unadjusted analysis, we also observed a significantly higher rate of head injuries among women compared with men, and in games compared with practices. However, sex-differences in the rate of head injuries appeared to vary significantly by the type of contact sustained. While there was insufficient evidence to suggest sex-differences in player-to-player contact resultant head injury rates, rates of head injuries from contact with apparatus, or with the playing surface were significantly higher in women than in men. This is difficult to explain, except possibly by sex-differences in playing style, technique, anatomy, or level of aggression. We explore the role of injury mechanism further, when evaluating sex-differences in the odds of concussion diagnoses, and time-loss resultant of head injuries. To our knowledge, we are the first to report the possibility that sex-differences in the odds of head injuries (given an injury), and in head injury corollaries (such as resultant concussions, and time-loss), may vary by the mechanism of injury (player-to-player contact) in this population. Future studies should evaluate the joint effect of sex and injury mechanism, on head injury rates and odds of head injuries, within other age-groups and player samples. Although we acknowledge the possibility of sex-differences in playing-style related contact exposure; based on the existing literature surrounding sex-differences in head injury outcomes, future studies may also consider the likelihood of physiological sex-differences in responses to the different biomechanical forces/loads imposed by varying contact exposures. We note that the multifactorial nature of head injury corollaries suggests the need for a similar approach when considering and developing injury prevention, and management programs as well.

We observed significant sex-differences in our examinations of concussions resultant of head injuries in this sample. As alluded to above, the observed sex-differences in rates of soccer-related head injuries and concussions have been reported previously (Agel et al. 2007; Covassin et al. 2003; Delaney et al. 2002; Dick et al. 2007b; Maher et al. 2014; Roos et al. 2016; Zuckerman et al. 2015), and possible explanations for this have been proposed. With respect to concussions, female athletes have been found to display greater concussion symptom reporting *intention*, than male athletes (Kroshus et al. 2017). From a physiological perspective, Delaney and colleagues (Delaney et al. 2002) suggest that a stronger neck and torso in male soccer players (relative to their female counterparts) could better dissipate the energy transmitted from head contact throughout the upper body, rather than having the skull or brain alone absorbing that impact (Aubry et al. 2002, Johnston et al. 2001). It has also been discussed that hormonal differences, differences in cerebral blood flow, or differences in glucose metabolism, may contribute to sex-differences in concussion susceptibility (Broshek et al. 2005; Covassin et al. 2012; Covassin et al. 2016). From our analyses, we see that sex-differences in the odds of concussion diagnosis from a head injury, are amplified by player-to-player contact mechanism. In fact, there is insufficient evidence to suggest sex-differences in the odds of concussion diagnosis, from head injuries resultant of other mechanisms. And so, in the future, it may be relevant to evaluate how specific injury mechanisms result in different physiological responses among male and female soccer players. Considering that we see strong evidence for the variability in sex-differences (by injury mechanism) on the multiplicative scale, future studies of concussion among soccer players may be directed toward examining the physiological sex-differences discussed above, within the context of biomechanical forces experienced from player-to-player contact. That is, it may be important to explore how sex-differences in neck musculature or cerebral blood flow, manifest in response to player-to-player contact in this setting. While it is difficult to draw conclusions based solely on the results seen here, it serves as a platform for these future examinations. It is important to explore these differences further (in different age-groups, and player samples), so that head injury management and rehabilitation plans are appropriately tailored for men and women.

We also observed significant sex-differences in our examinations of time-loss resultant of head injuries in this sample. There exists evidence to suggest that female collegiate soccer players experience greater mean time-loss due to concussions, as compared with their male counterparts (Covassin et al. 2016). However, we are the first to evaluate sex-differences in time-loss from all head injuries, after accounting for several other important covariates. Moreover, we are also the first to report that these sex-differences, may vary by injury mechanism. In our analyses, time-loss from non-player-to-player contact mechanisms (such as contact with apparatus, surface etc.) appears to be significantly greater in female players. At the same time, there is insufficient evidence in our analyses to suggest sex-differences in time-loss from player-to-player contact resultant head injuries. It is possible to explain sex-differences in time-loss from head injuries resulting in concussions. Indeed, there are data to indicate that following an acute concussion, women perform worse on neuropsychological tests and report more symptoms of concussion than do men (Broshek et al. 2005; Colvin et al. 2009; Zuckerman et al. 2012). Broshek et al. (Broshek et al. 2005) observed significant sex-differences in the decline of both simple and complex reaction times following a concussion in a large sample of high school and college athletes. Moreover, these sex-differences were apparent with regard to both the degree of cognitive decline from pre-season and the frequency of impaired cognitive performance and were independent of a number of co-varying factors such as age and injury severity. However, it is important to recognize that we observed sex-differences in time-loss resultant of all head injuries. Moreover, we observed evidence for variability in these sex-differences across levels of injury mechanism, on a multiplicative scale. With respect to time-loss, it may be relevant to build on the physiological sex-differences discussed above, and evaluate such differences in the context of biomechanical loads experienced from head contact with the ball, goalpost, or the playing surface.

We note several limitations to this analysis. First, the low participation in reporting among NCAA programs limits both the stability of the estimates and the external validity of the findings. Due to the nature of the surveillance system, we are unable to identify the unique number of teams, and athletes contributing data to it. Moreover, injury and exposure data were collected as event-based counts, and therefore there is no way to link head injury data to individual characteristics (e.g., age, years of playing experience) or to environmental or policy changes, in order to directly test etiologic hypotheses. Furthermore, collecting exposures as event based counts prohibits us from computing person-time more precisely (in h/min player), and this limits our understanding particularly with respect to game-related injury incidence. Also, the NCAA-ISS does not consider injuries that occur outside of NCAA-sanctioned events and therefore may have underestimated the full scope of soccer injuries among these players. Due to the structure of the surveillance system, we are also unable to track multiple head injuries to the same player, on a specific timeline. With respect to head injuries, we also acknowledge the limitations posed by examining a dichotomously defined concussion diagnosis (as a measure of head injury severity), due to the transient nature of concussion definitions and from what is known regarding the significance of symptom burden in determining severity, and recovery from concussions (Meehan et al. 2014). Finally, the inferences from our models are limited to the injured cohort, rather than to the entire player population.

These limitations notwithstanding, the NCAA-ISS provides the most comprehensive set of injury-related data to date among collegiate athletes in the United States. Although these data are useful for generating hypotheses, more comprehensive data collection on sociodemographic characteristics and the specific determinants of injury, coupled with greater participation among NCAA programs would greatly improve our understanding of head injury occurrence within this population.

## Conclusions

We observed that sex-differences in head injury incidence, odds of head injuries within the injured sample, and in head injury corollaries, varied by injury mechanism. Based on the results discussed here, it is reasonable to suspect that unidimensional (or identical) injury prevention and management programs may not be equivalently effective for male and female players. And so, these results may be used as a platform to not only inform future studies, but also to better tailor injury prevention and management strategies for male and female players. Given the prominence of soccer play in the United States, public health efforts should promote the use of this surveillance system to better inform, evaluate, and improve head injury prevention practices. Policies for concussion management and return-to-play must consider sex-differences in injury likelihood and specific injury mechanisms, rather than only relying on uniform guidelines.
